# Replicating Adenovirus-SIV Immunization of Rhesus Macaques Induces Mucosal Dendritic Cell Activation and Function Leading to Rectal Immune Responses

**DOI:** 10.3389/fimmu.2019.00779

**Published:** 2019-04-12

**Authors:** Eun-Ju Ko, Sabrina Helmold Hait, Gospel Enyindah-Asonye, Mohammad Arif Rahman, Tanya Hoang, Marjorie Robert-Guroff

**Affiliations:** Vaccine Branch, National Cancer Institute, National Institutes of Health, Bethesda, MD, United States

**Keywords:** dendritic cells, adenovirus-recombinant vaccine, rhesus macaque, simian immunodeficiency virus, mucosal immunity, rectal mucosa, T and B cell memory responses

## Abstract

Inducing strong mucosal immune responses by vaccination is important for providing protection against simian immunodeficiency virus (SIV). A replicating adenovirus type 5 host range mutant vector (Ad5hr) expressing SIV proteins induced mucosal immune responses in rectal tissue associated with delayed SIV acquisition in female rhesus macaques, but the initial mechanisms leading to the induced immunity have not been elucidated. As dendritic cells (DCs) are known to orchestrate both innate and adaptive effector immune cell responses, we investigated their role here. Rhesus macaques were immunized twice mucosally with a replicating Ad5hr expressing SIV Env, Gag, and Nef (Ad-SIV) or empty Ad5hr vector (Ad-Empty). DC subsets and their activation were examined in rectal tissue, blood, and LNs at 3 timepoints after each immunization. Plasmacytoid DCs, myeloid DCs, and Langerhans cells were significantly increased in the rectal mucosa, but only myeloid DCs were significantly increased in blood post-immunizations. All rectal DC subsets showed increased frequencies of cells expressing activation markers and cytokines post-immunization, blood DCs showed mixed results, and LN DCs showed few changes. Rectal DCs responded strongly to the vector rather than expressed SIV antigens, but rectal DC frequencies positively correlated with induced rectal antigen-specific memory T and B cells. These correlations were confirmed by *in vitro* co-cultures showing that rectal Ad-SIV DCs induced proliferation and antigen-specific cytokine production by autologous naïve T cells. Our results highlight the rapid response of DCs to Ad immunization and their role in mucosal immune activation and identify initial cellular mechanisms of the replicating Ad-SIV vaccine in the rhesus macaque model.

## Introduction

The mucosal immune barrier is critical for protection against human immunodeficiency virus (HIV) infection because most HIV transmissions occur across mucosal surfaces (1, 2). The initial barrier includes mucus and IgA which can trap virus particles and prevent virus invasion (3, 4). If this mucosal barrier is breached, the virus can access cells in the underlying mucosa. Thereafter, binding of the virus to CD4 and co-receptors can lead to infection of macrophages and T cells, in some cases facilitated by binding to the integrin α4β7 (5). Binding to DC-SIGN on dendritic cells (DCs) can result in viral transport to local lymphoid tissue leading to infection of CD4^+^ T cells (3, 6–9). Therefore, strong mucosal immunity is important for protective efficacy of HIV vaccines.

While replication-incompetent adenoviruses (Ad) are being developed as HIV vaccine vectors (10–12), our approach uses replication-competent Ad-recombinants based on the rationale that replicating vaccines are highly effective and elicit life-long immunity. Examples are numerous, including vaccines for small pox, polio, measles, and yellow fever virus (13). Additionally, Ad target cells at mucosal sites, and therefore elicit mucosal immunity. Our overall vaccine approach involves Ad-recombinant mucosal priming followed by systemic boosting with viral envelope proteins. This strategy showed protective efficacy initially in chimpanzee HIV models, and later in rhesus macaque simian immunodeficiency virus (SIV) and SHIV models, resulting in movement of the approach to human clinical trials (14). Overall protective effects have been associated with multiple immune responses including strong binding antibody, neutralizing and non-neutralizing antibodies, memory B cells, antigen-specific CD4^+^ and CD8^+^ T cell responses in local tissues and blood and antigen-specific T follicular helper cells in lymph nodes (LN) (14–20). Of relevance for the studies here, delayed acquisition of SIV infection following immunization by our prime/boost approach was correlated with localized mucosal IgA immunity (21). A subsequent study documented delayed SIV acquisition in vaccinated female but not male rhesus macaques, associated with Env-specific rectal IgA and memory B cells, and total rectal plasma cells (22). However, factors associated with Ad vaccine-induced immunity at the rectal site have not been fully elucidated.

DCs are potent antigen presenting cells orchestrating both innate and adaptive responses by producing pro-inflammatory cytokines, transporting antigens to draining LNs, and presenting antigen to CD4^+^ T cells. Subsequent antigen-specific antibody production by B cells and cytotoxic CD8^+^ T cell responses are dependent on the resultant CD4^+^ T cell help provided. DCs can also induce cytotoxic CD8^+^ T cell activation directly by cross-presentation. Since DCs are located within or beneath the epithelial lining of mucosal tissues, they are one of the first immune cells to encounter pathogens and immunogens (23). There are two distinct types of DCs. Plasmacytoid DCs (pDCs) are known as type 1 interferon (IFN) producing cells and play a role in antiviral activity. Myeloid DCs (mDCs) express the typical myeloid marker, CD11c. They can detect pathogens by pathogen pattern recognition receptors including toll-like receptor (TLR) 2 and 4. Langerhans cells (LCs), a specialized mDC population found in the epithelium, are distinguished by high CD1a and langerin expression. Overall, DCs recognize pathogens and immunogens, initiate innate immune responses, and elicit adaptive immunity (24–26). Therefore, they are an important target cell population for successful vaccine development. Understanding their activation and function after immunization by various regimens should aid vaccine design (27).

Previously using a rhesus macaque model, we demonstrated that following mucosal immunization replicating Ad type 5 host range mutant (Ad5hr)-SIV recombinants express their inserted transgene at least 25 weeks. This expression persisted in mucosal macrophages and mDCs (28). However, the DC subsets which were recruited and activated by the vector at the mucosal tissues and responsible for inducing adaptive immune responses were not identified. Here, we investigated mucosal and systemic DC activation and function after replicating Ad5hr-SIV mucosal immunizations of rhesus macaques to elucidate early responses leading to both cellular and humoral mucosal immunity.

## Materials and Methods

### Animals, Immunization and Sample Collection

**Figure 1:**
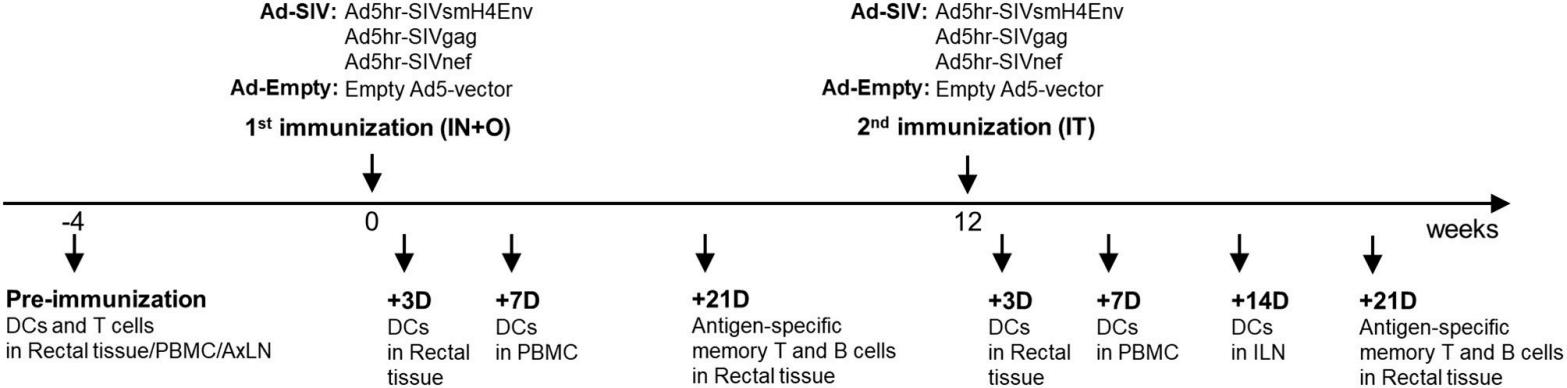
Immunization and sample collection schedule. The immunization was given two times mucosally with a 12-weeks interval. Rectal biopsies were collected before and at day 3 and 21 post immunizations, Blood was collected before and at day 7 post immunizations. Axillary LNs were collected before the immunization and Inguinal LNs were collected at day 14 after the 2nd immunization.

Female rhesus macaques were immunized at week 0 (intranasally and orally) and week 12 (intratracheally) with replicating Ad5hr recombinants separately expressing SIV_smH4_*env*, SIV*gag*, and SIV*nef* (*n* = 38, Ad-SIV) at a dose of 5 × 10^8^ plaque forming units/recombinant/site or with Ad5hr empty vector (*n* = 22, Ad-Empty) at a dose equivalent to the Ad-SIV recombinants administered. All rhesus macaques were maintained at the NCI animal facility under the guidelines of the Association for the Assessment and Accreditation of Laboratory Animal Care. The protocols and procedures were approved by the NCI Animal Care and Use Committee. Immunization and sample collection schedules are shown in Figure 1. Rectal biopsies were obtained from macaques (20 Ad-SIV immunized, 10 Ad-Empty controls) before, at day 3 and day 21 after each immunization. The biopsies were digested with collagenase (2 mg/ml, Sigma Aldrich) and single cells were harvested as described (29). Blood samples were collected before and at day 7 post-immunizations (18 Ad-SIV immunized, 12 Ad-Empty controls). PBMCs were prepared by centrifugation over Ficoll gradients as described (30). Axillary LN biopsies were collected before immunization, and inguinal LN biopsies were obtained at day 14 after the 2nd immunization (19 Ad-SIV immunized, 11 Ad-Empty controls). Biopsies were minced and passed through a 40 μm cell strainer to obtain single cells as described (20). PBMCs and LN cells were frozen in fetal bovine serum containing 10% DMSO and stored in liquid nitrogen until use. Rectal cells were used fresh immediately after the harvest.

### Flow Cytometry

Rectal cells, PBMC, and LN cells were stained with Aqua Live/Dead viability dye (Invitrogen), V500 anti-CD3 (SP-34), and BV510 anti-CD56 (B159) (BD Biosciences), and BV510 anti-CD20 (2H7) (Biolegend) to exclude dead and lineage^+^ cells. DCs were phenotyped by staining with BV711 anti-CD11c (3.9), Alexa Fluor 700 anti-HLA-DR (L243), Alexa Fluor 488 anti-CD14 (M5E2), and BV605 anti-CD1c (L161) (Biolegend) and BV412 anti-CD123 (7G3) (BD Biosciences). For analysis of DC activation markers, the cells were stained with the DC phenotyping antibodies plus PE-Cy5 anti-CD40 (5C3) and Biotin anti-CD86 (IT2.2) (BD Biosciences), PE-eFluor610 streptavidin, and PerCP-eFluor710 anti-CCR7 (3D12) (eBioscience). For DC cytokine production, the cells were incubated with BD Golgi-stop for 6 h and stained with DC surface phenotyping antibodies. Then the cells were stained with PE anti-interleukin (IL)-6 (MQ2-6A3) and PE-Cy7 anti-tumor necrosis factor (TNF)-α (MAB11) (BD Biosciences) and APC anti-B cell activating factor (BAFF) (1D6) (eBioscience) after fixation and permeabilization with a Cytofix/Cytoperm Kit (BD Biosciences). For intracellular cytokine staining of rectal T cells, cells obtained 21 days post-immunization were cultured with BD Golgi-stop, ECD anti-CD28 (CD28.2) (Beckman Coulter), purified CD49d (9F10) (eBioscience), and BV605 anti-CD4 (OKT4) (Biolegend). The cells were also stimulated with pooled peptides at 1 μg/ml final concentration representing SIV_mac239_ Gag (AIDS Research and Reference Reagent Program), SIV_M766_ gp120 Env (Advanced BioScience Laboratories, Inc., Rockville, MD; ABL) or SIV_CG7V_ gp120 Env (ABL). The pools consisted of 15-mer peptides overlapping by 11 amino acids. After 6 h incubation, the cells were stained with Aqua Live/Dead viability dye, BV786 anti-CD3 (SP34.2) (BD Biosciences), eVolve655 anti-CD8 (RPA-T8), PE-CY5 anti-CD95 (DX2) (eBioscience), and then intracellularly stained following fixation/permeabilization with FITC anti-TNF-α (MAB11) (BD Bioscience), Alexa Fluor 700 anti-interferon (IFN)-γ (B27) (Life Technologies), and APC-Cy7 anti-IL-2 (MQ1-17H12) (Biolegend). For antigen-specific memory B cell staining, rectal cells obtained 21 days post-immunization were stained with biotinylated SIV_CG7V_, APC streptavidin, Alexa Fluor 700 anti-CD3 (SP34-2), BV650 anti-CD20 (2H7), BV605 anti-CD21 (B-ly4) (BD Biosciences), PE-Cy5 anti-CD19 (J3-119) (Beckman Coulter), and PE-TxRed anti-IgD (AT-1) (StemCell). The stained cells were acquired on an LSR II (BD Biosciences) and analyzed using FlowJo software.

### DC Functional Assay

Eight macaques from the Ad-SIV group and five macaques from the Ad-Empty group were used in co-culture experiments. Naïve autologous T cells were enriched using a pan T cell isolation kit (Miltenyi Biotech) from PBMC obtained prior to immunization (Supplementary Figure 1A). T and B cells were excluded from rectal cells by negative selection using anti-non-human primate (NHP) CD3 and CD20-microbeads (Miltenyi Biotech). Rectal DCs were then positively selected using biotin anti-CD123 (7G3) for pDCs, or biotin anti-CD11c (3.9) for mDCs plus anti-biotin microbeads (Supplementary Figure 1B). DCs (1 × 10^4^ cells/well) and T cells (1 × 10^5^ cells/well) were co-cultured in a U-bottom 96-well plate for 6 days. For the T cell proliferation assay, naïve T cells were stained with 3 μM of carboxyfluorescein succinimidyl ester (CFSE, Sigma Aldrich) before co-culture and CFSE^low^CD3^+^CD4^+^ or CFSE^low^CD3^+^CD8^+^ cells were analyzed. The proliferated cell percentages are reported following subtraction of CFSE^low^CD3^+^CD4^+^ or CFSE^low^CD3^+^CD8^+^ values in co-cultures lacking DC. For cytokine assays, T cells were re-stimulated with or without Env peptide pools (SIV_M766_ plus SIV_CG7V_) at a 0.5 ug/ml final concentration plus BD Golgi-stop for 6 h after 6 days co-culture and then surface stained with Aqua Live/Dead viability dye, BV786 anti-CD3 (SP34.2), eVolve655 anti-CD8 (RPA-T8), and BV605 anti-CD4 (OKT4). Intracellular staining following fixation/permeabilization was then conducted as above for the rectal T cells. SIV Env-specific cytokine production of T cells was reported following subtraction of cytokine positive values in wells lacking peptide.

### Statistical Analysis

DC frequencies and activation in different groups at different times were analyzed by two-way ANOVA with the Tukey multiple-comparison test. Correlations were assessed using the Spearman rank correlation test and co-culture data were analyzed by unpaired *t*-test. GraphPad Prism was used for statistical analysis.

## Results

### Rectal pDCs and LCs Increase and Become Activated After Mucosal Ad5hr Immunizations

**Figure 2:**
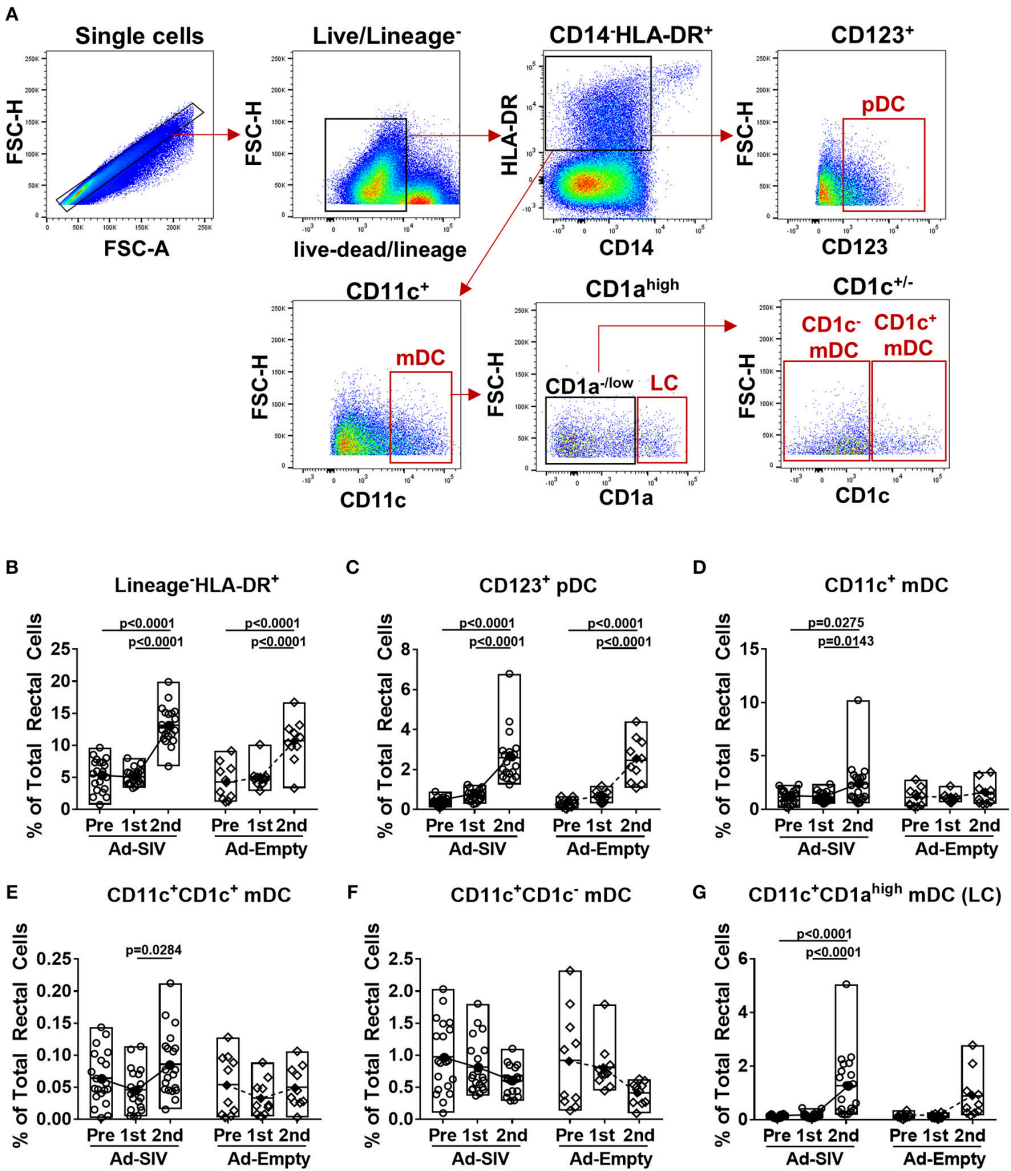
DCs are recruited to the rectal mucosa within 3 days of mucosal replicating Ad5hr immunization. Rhesus macaques received two mucosal immunization with Ad-SIV or Ad-Empty at a 12-weeks interval and rectal biopsies were collected before and 3 days after each immunization. **(A)** Gating strategy of rectal DCs. **(B–G)** Percentages of each DC population in total rectal cells. The open and filled dots show the individual and mean values, respectively. For statistical analysis, two-way ANOVA and Tukey's post-multiple comparison test were performed.

To identify DC subsets recruited and activated at the rectal mucosa after Ad5hr immunizations, we collected rectal biopsies before and 3 days post-immunizations. Rectal DCs were discriminated by gating CD123^+^ (pDC), CD11c^+^ (mDC), CD1a^high^CD11c^+^ (LC), CD1c^+^CD11c^+^ (CD1c^+^ mDC), and CD1c^−^CD11c^+^ (CD1c^−^ mDC) in the live lineage^−^CD14^−^HLA-DR^+^ population (Figure 2A). Note that an antibody generally used to identify LC in humans, anti-CD207 (which is also known as langerin), is not available for rhesus macaques. Before immunization, frequencies of pDCs and mDCs were <0.5% and ~1% of total rectal cells, respectively. Compared to pre-immunization, lineage^−^ CD14^−^HLA-DR^+^ cell populations were significantly increased (2.4-fold in Ad-SIV and 2.5-fold in Ad-Empty groups) after the 2nd immunization (*p* < 0.0001 for both; Figure 2B). The pDC population was significantly increased (7.0-fold in Ad-SIV and 8.2-fold in Ad-Empty groups, *p* < 0.0001 for both after the 2nd immunization; Figure 2C). Total CD11c^+^ mDC and CD11c^+^CD1a^high^ LCs frequencies were significantly increased in the Ad-SIV macaques (2.1-fold and 11.4-fold, respectively) and tended to increase in the Ad-empty macaques (Figures 2D,G). The CD11c^+^CD1c^+^ mDC population exhibited little change (Figure 2E), but CD11c^+^CD1c^−^ mDCs frequencies were decreased following the 2nd immunization in both immunized and control macaques (Figure 2F). Before immunization, the mean rectal mDC frequency was 1.17% (Figure 2D), higher than the mean pDC frequency (0.37%, Figure 2C). However, after the 2nd immunization, the mean rectal pDC frequency was highly increased to 2.6% of total rectal cells (Figure 2C), higher than the mean mDC frequency (2.4%, Figure 2D). These data suggest that LC within the epithelial lining and pDCs which expressed TLR7 quickly recognized the Ad5hr immunogen and expanded at the rectal mucosa. Overall, there were no differences in DC frequencies between Ad-SIV and Ad-Empty groups, indicating that DC recruitment was caused by the vector itself, not by the expressed SIV antigens.

**Figure 3:**
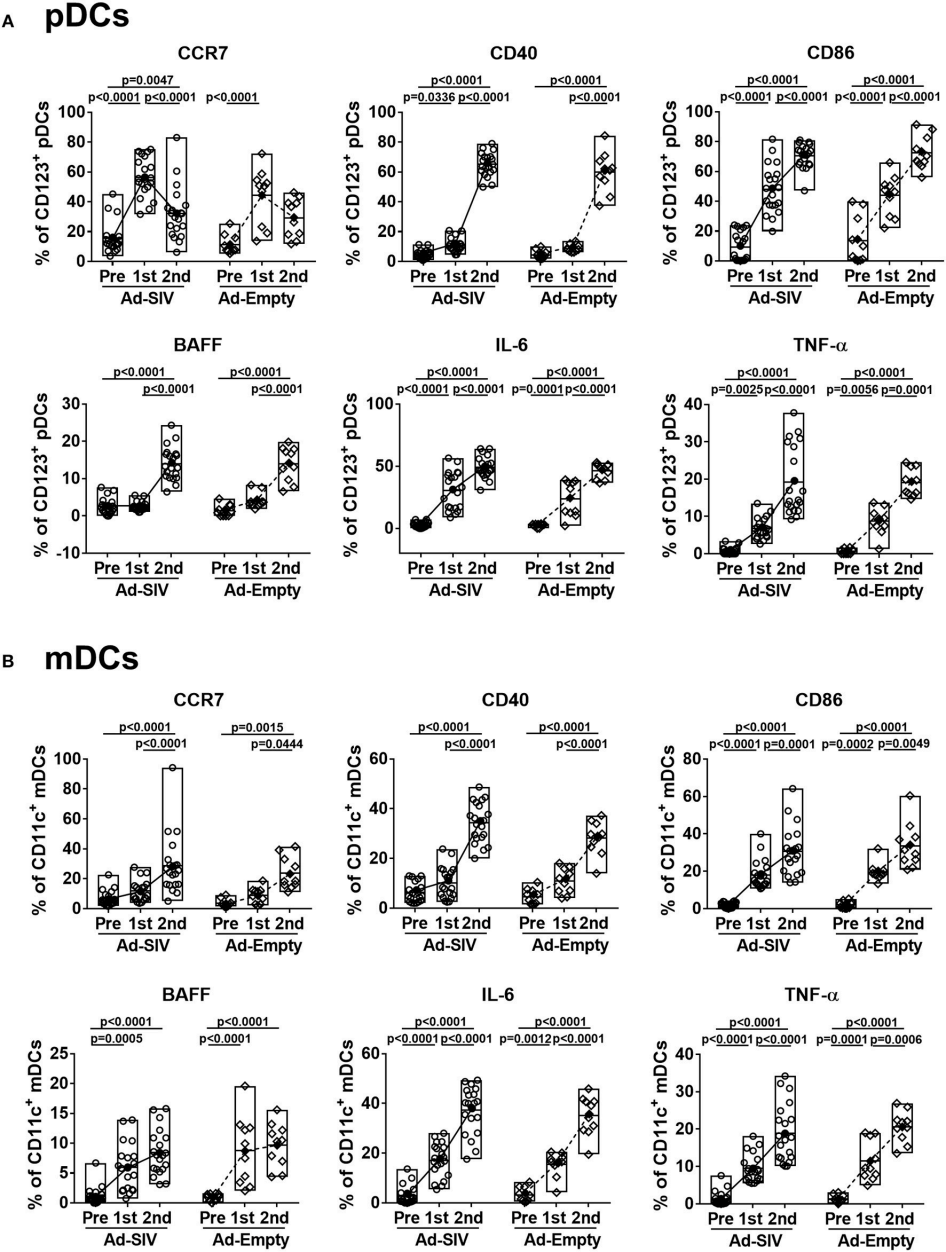
Mucosal Ad5hr immunization induces rectal DC activation. The frequencies of rectal pDCs **(A)** and mDCs **(B)** expressing migration and activation markers and cytokines were measured before and at day 3 after each immunization. The open and filled dots show the individual and mean values, respectively. For statistical analysis, two-way ANOVA and Tukey's post-multiple comparison test were performed.

When DCs take up antigen at local tissue sites, they undergo activation and migrate to draining LNs for antigen presentation to naïve CD4 T cells. Activated DCs also produce cytokines to stimulate other immune cells. Upregulation of CCR7 on DCs signals migration to lymphoid tissue (31). After the 1st Ad immunization, CCR7^+^ pDC frequencies in both Ad-SIV and Ad-Empty groups were significantly increased. They declined after the 2nd immunization but remained higher compared to pre-immunization levels (Figure 3A). In contrast, CCR7^+^ mDC frequencies gradually increased during the immunizations (Figure 3B). Thus, both pDC and mDC in the rectal mucosa showed increased migratory potential after the Ad immunizations. CD40 and CD86 are DC co-stimulatory and activation markers. Frequencies of both pDCs and mDCs expressing each of the molecules were increased following the Ad immunizations, with significant differences compared to pre-immunization values (Figure 3). BAFF is an important cytokine for B cell proliferation, differentiation and survival (32, 33). Percentages of BAFF^+^ pDCs significantly increased after the 2nd Ad immunization, whereas BAFF^+^ mDC frequencies were gradually increased after both Ad immunizations (Figure 3). Frequencies of DCs expressing the pro-inflammatory cytokines, IL-6 and TNF-α, were also gradually increased during the immunizations in both pDCs and mDCs with high significance (Figure 3). Similar to overall DC frequencies, there were no differences between Ad-SIV and Ad-Empty groups in frequencies of DC expressing various activation markers or cytokines.

These data suggest that rectal pDCs and LCs were most responsive to the replicating Ad vectors. However, both pDCs and mDCs quickly recognized the replicating Ad, with increased percentages of cells expressing co-stimulatory molecules, migration markers and cytokines. All these responses tended to be higher in pDCs compared to mDCs, suggesting rectal pDCs might be more responsive to replicating Ad5hr exposure.

### Mucosal Ad Immunizations Increase mDCs, but Activate Both pDCs and mDCs in Blood

**Figure 4:**
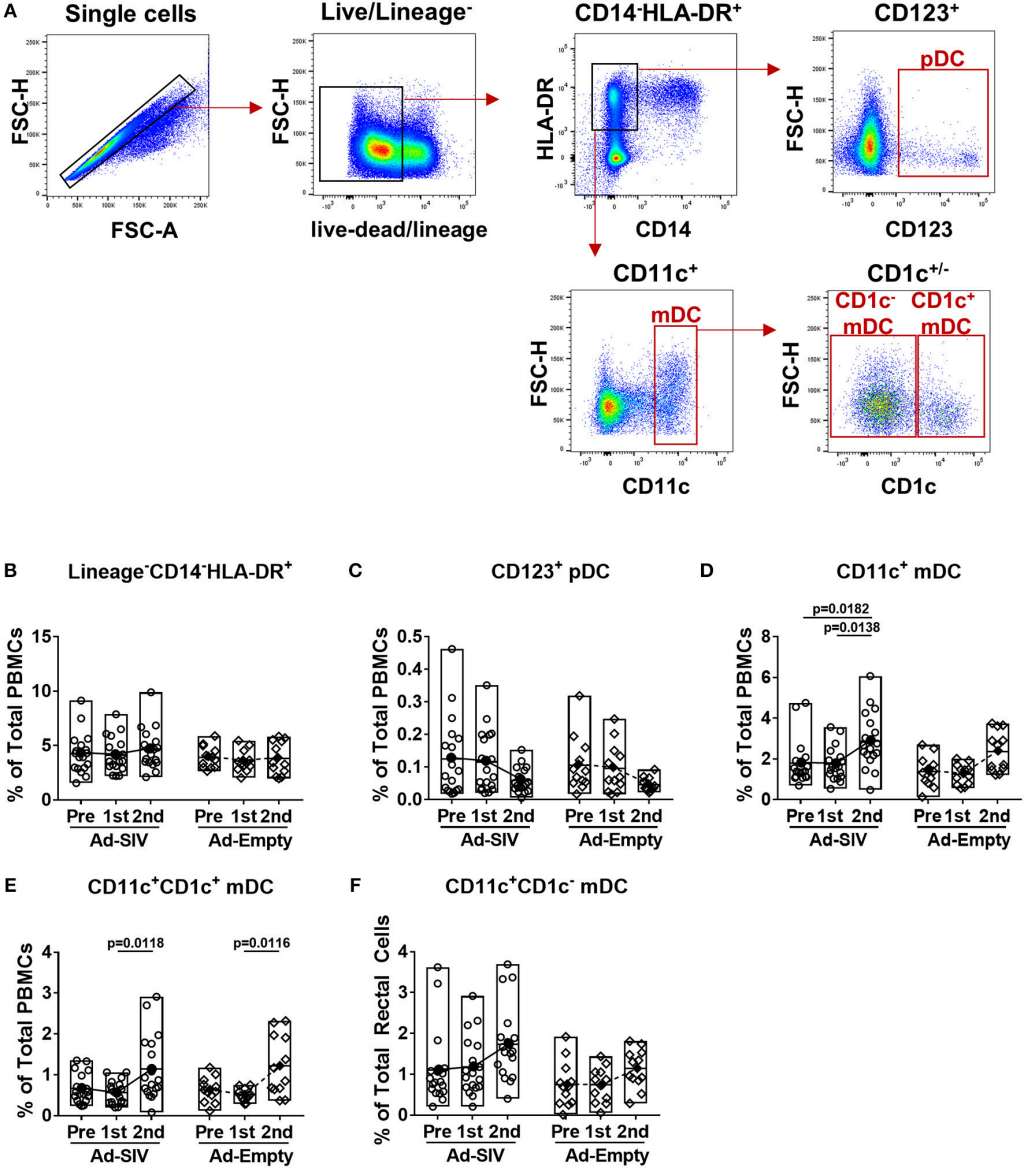
Myeloid DCs are increased in blood 7 days after mucosal replicating Ad5hr immunization. Blood samples were collected from the Ad-SIV and Ad-empty immunized rhesus macaques before and 7 days after each mucosal immunization. **(A)** Gating strategy of blood DCs. **(B–F)** Percentages of each DC population in total PBMCs. The open and filled dots show the individual and mean values, respectively. For statistical analysis, two-way ANOVA and Tukey's post-multiple comparison test were performed.

Circulating DC frequencies and activation after mucosal Ad immunizations were evaluated using blood samples collected at day 7 following each immunization. DC subsets were discriminated by gating of CD123^+^, CD11c^+^, and CD1c^+/−^ in the live lineage^−^CD14^−^HLA-DR^+^ population (Figure 4A). Overall the lineage^−^CD14^−^HLA-DR^+^ cell population was approximately 4–5% in total PBMCs and did not change post-immunizations (Figure 4B). Frequencies of pDCs showed decreased trends over the course of immunization (Figure 4C), but those of mDCs significantly increased from 2 to 4% after the 2nd immunization (Figure 4D). Both CD1c^+^ and CD1c^−^ mDCs also increased after the 2nd immunization (Figures 4E,F). As with rectal DCs, no significant differences were observed between Ad-SIV and Ad-Empty groups.

**Figure 5:**
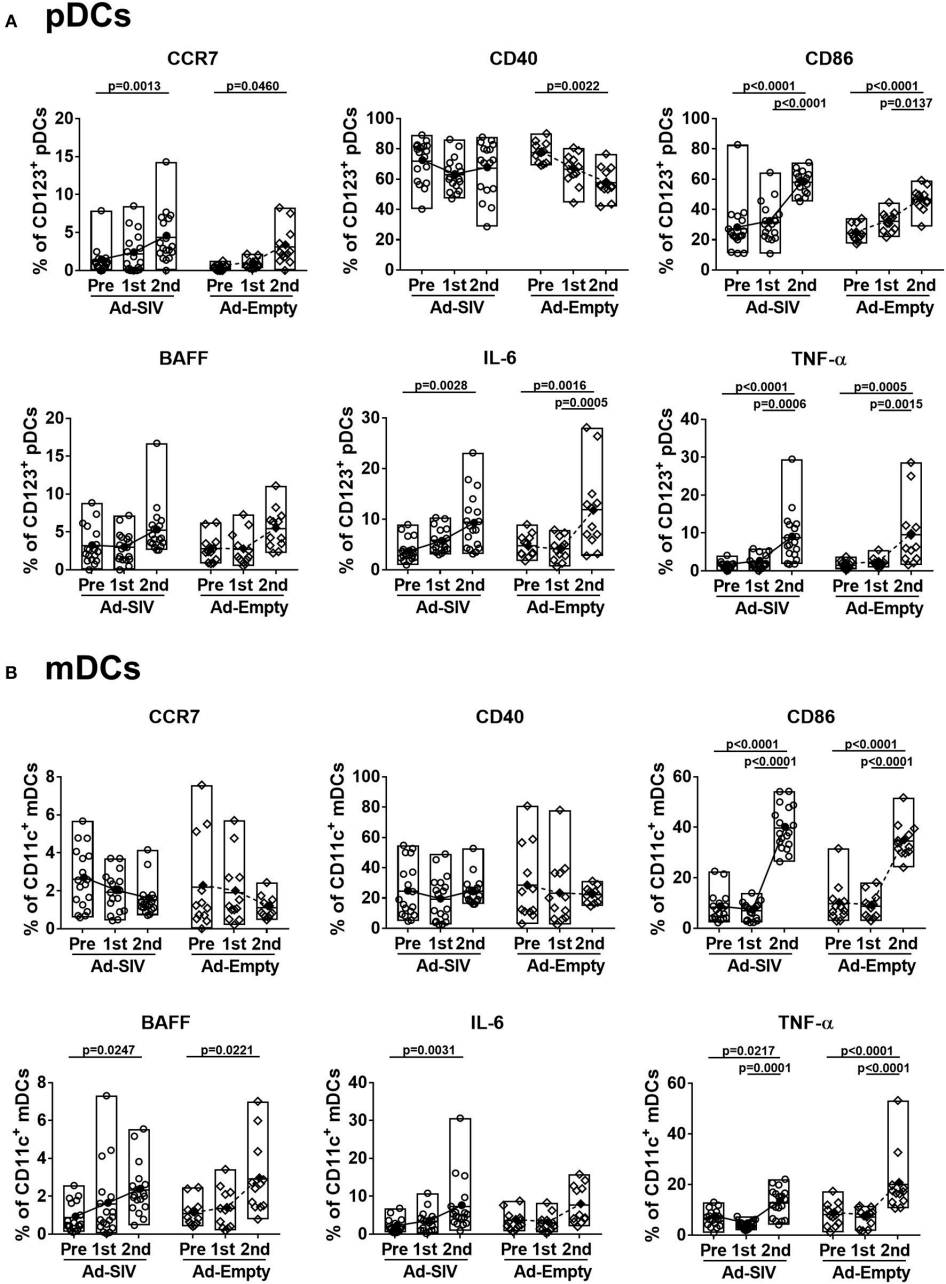
Mucosal Ad5hr immunization induces blood DC activation. The frequencies of blood pDCs **(A)** and mDCs **(B)** expressing migration and activation markers, and cytokines were measured before and at day 7 after each immunization. The open and filled dots show the individual and mean values, respectively. For statistical analysis, two-way ANOVA and Tukey's post-multiple comparison test were performed.

Although the mean percentages of pDCs in blood over all three time points (0.06–0.13% of total PBMCs, Figure 4C) were lower than those in rectal mucosa over the same time points (0.4–2.7% of total rectal cells, Figure 2C) and also lower compared to circulating mDC (1.8–2.9% of total PBMCs, Figure 4D), activation of blood pDCs was significant after mucosal immunization (Figure 5A). Frequencies of circulating CCR7^+^, CD86^+^, IL-6^+^, and TNF-α^+^ pDCs were significantly increased after the 2nd immunization, and BAFF^+^ pDCs also increased although not significantly. High CD40^+^ pDC percentages pre-immunization were maintained in the Ad-SIV group but decreased in the Ad-Empty group after the 2nd immunization. While the frequency of mDCs in blood was increased after the 2nd immunization, the frequency of CCR7^+^ mDCs tended to decrease, and CD40^+^ mDC frequencies were unchanged (Figure 5B). Frequencies of the other cytokine^+^ mDCs were enhanced after the 2nd immunization. Overall, blood DCs appeared to be more activated prior to immunization compared to rectal DCs.

### Little Effect of Mucosal Ad Immunizations on LN DCs

**Figure 6:**
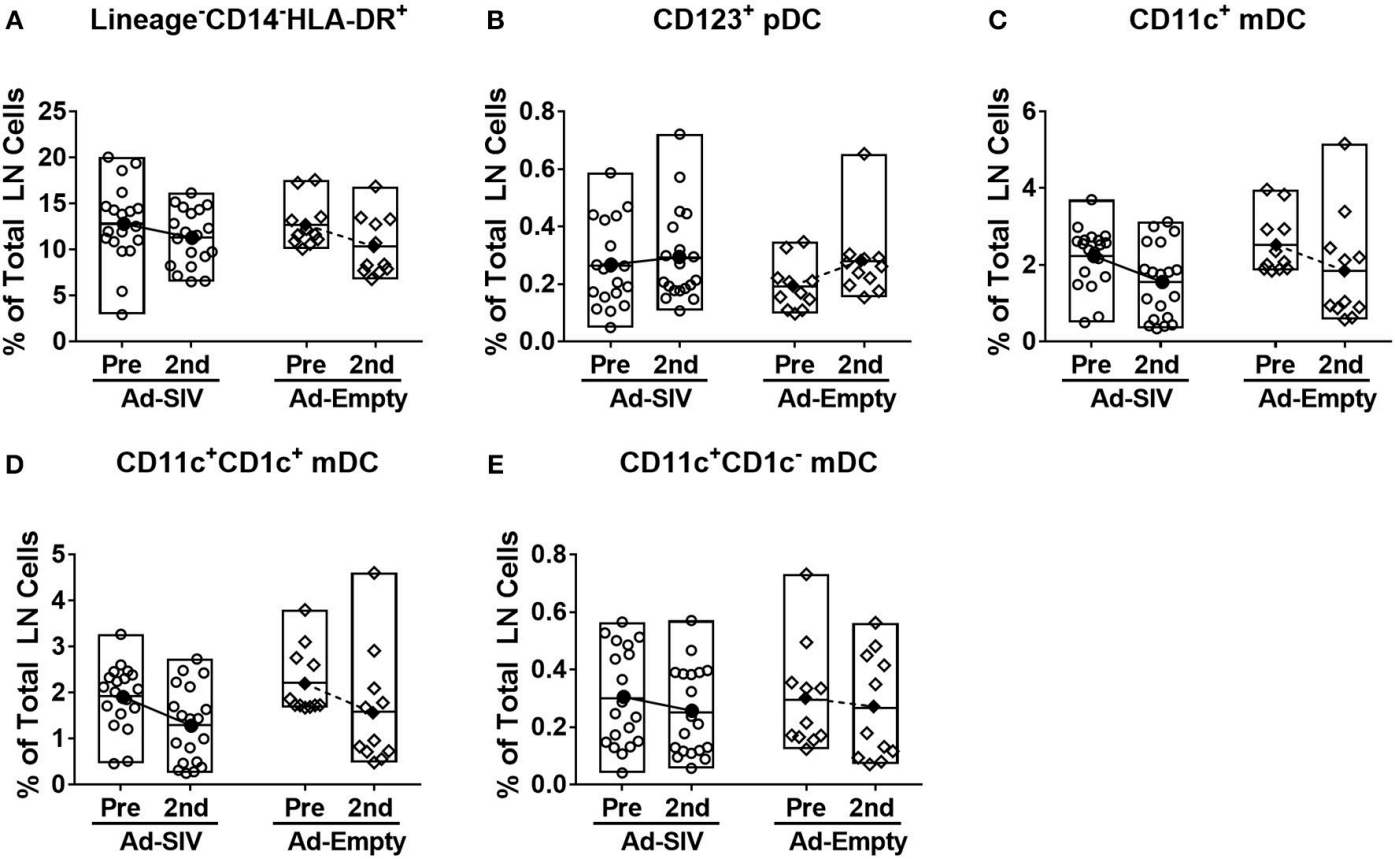
Mucosal Ad5hr immunizations do not affect the frequency of LN DC populations. Axillary LNs were collected before immunization and inguinal LNs were collected at day 14 after the 2nd immunization. **(A–E)** Percentages of each DC population in total LN cells. The open and filled dots show the individual and mean values, respectively. For statistical analysis, two-way ANOVA and Tukey's post-multiple comparison test were performed.

**Figure 7:**
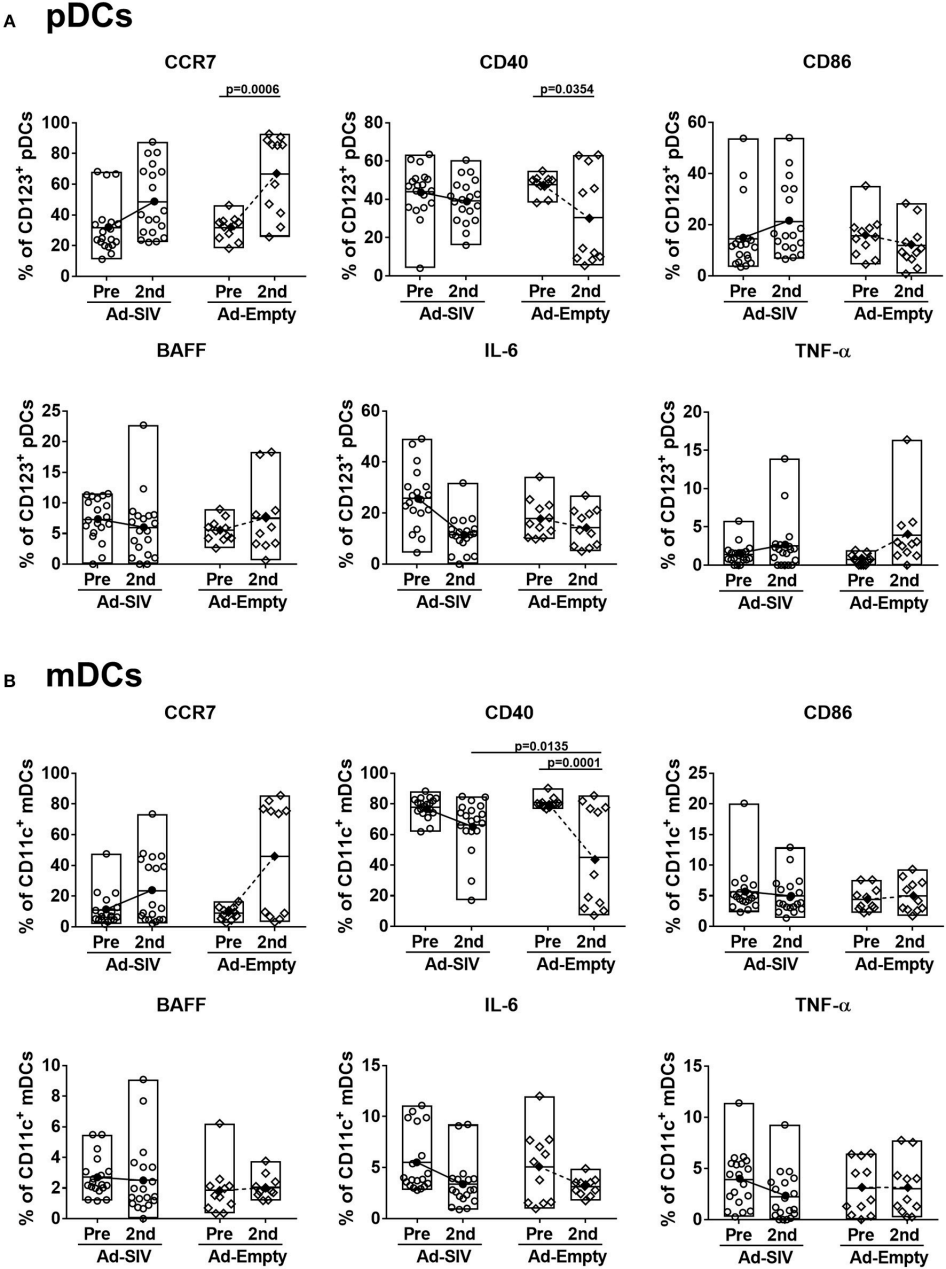
Migration marker expression is increased in LN DCs. The frequencies of LN pDCs **(A)** and mDCs **(B)** expressing migration and activation markers, and cytokines were measured before and at day 14 after the 2nd immunization. The open and filled dots show the individual and mean values, respectively. For statistical analysis, two-way ANOVA and Tukey's post-multiple comparison test were performed.

After antigen uptake and maturation DCs migrate to LNs to initiate adaptive immune responses. LN biopsies were collected before immunization and 14 days after the 2nd immunization. The LN DC gating strategy was the same as for blood (Figure 4A). In LNs, prior to immunization lineage^−^CD14^−^HLA-DR^+^ cells were ~5–20% of total LN cells (Figure 6A), but the mean pDC population was only 0.25% (Figure 6B), and mDCs were approximately 2% (Figure 6C). The pDC frequencies showed a slight increasing trend while CD1c^+^ and CD1c^−^ mDC and total mDC frequencies were decreased after immunization, but no significant differences were seen (Figures 6B–E). Regarding activation and cytokine expression of LN DCs (Figure 7), only the frequencies of CCR7^+^ pDCs and mDCs increased post-immunization, significantly for the former in the Ad-empty group but not the latter. DC populations expressing other activation markers showed no changes aside from a significant decrease in the CD40^+^ pDC and mDC populations of the Ad-empty group post-immunization. These results were mirrored by changes in the DC subsets when expression of each activation marker and cytokine was examined. The mean fluorescence intensity (MFI) of CCR7^+^ pDCs and mDCs increased significantly following the 2nd immunization in the Ad-empty group, while the MFI of CD40^+^ pDCs and mDCs decreased (Supplementary Figure 2). Similar to blood DCs, LN DCs showed higher frequencies of CD40^+^ cells than rectal DCs prior to immunization, indicating that LN DCs generally maintained an activated status.

### Rectal DC Frequencies Correlate With Antigen-Specific Rectal T and B Cell Responses

**Figure 8:**
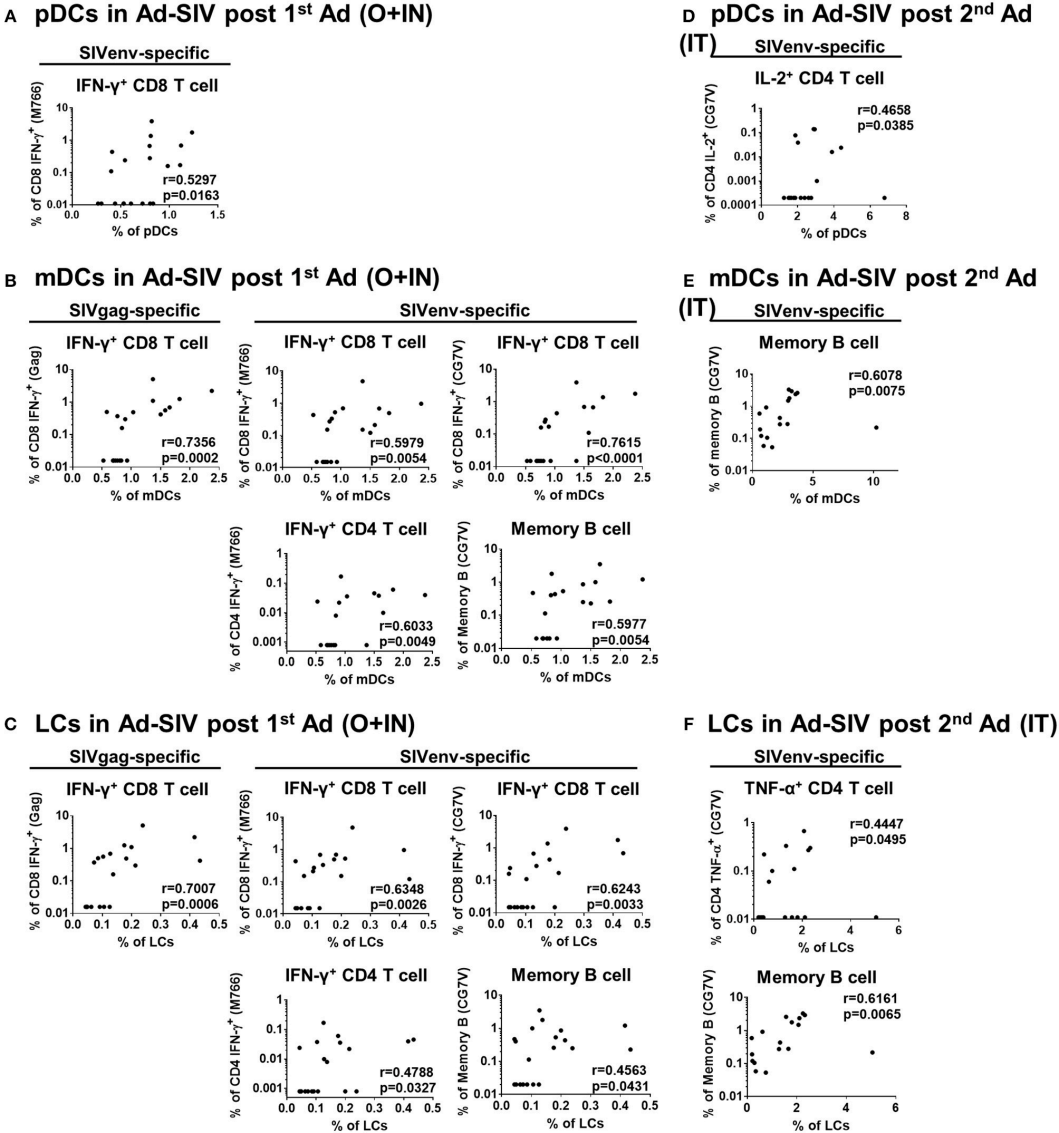
DC frequencies in Ad-SIV immunized group are positively correlated with antigen-specific T and B cell responses in rectal mucosa. Non-parametric Spearman test was performed for correlation analysis by using the data obtained after 1st Ad-SIV **(A–C)** and 2nd Ad-SIV **(D–F)** immunizations. Frequencies of Ad-SIV immunized rectal DCs were measured at day 3 after each immunization. Rectal antigen-specific T and B cell responses in Ad-SIV group were measured at day 21 after each immunization. SIV Gag, SIV_M766_ Env, or SIV_CG7V_ Env peptide pools as indicated were used for 6 h of T cell stimulation. The antigen-specific responses are reported following subtraction of unstimulated values.

Mucosal DCs induce antigen-specific T cell and antibody producing plasma cell homing to the mucosa (34, 35). To investigate this connection between DCs and adaptive immune cells, we performed correlation analyses between frequencies of rectal DC populations 3 days post-immunizations in the Ad-SIV immunized group and those of rectal antigen-specific cytokine producing T cells and memory B cells 21 days post-immunization. Positive correlations were observed, especially with CD8^+^ memory T cells (Figure 8). Correlation analyses between rectal pDCs after the 1st Ad-SIV immunization and SIV-specific IFN-γ-producing rectal CD8^+^ memory T cells (CD3^+^CD8^+^CD95^+^IFN-γ^+^) showed a positive correlation (*r* = 0.5297, *p* = 0.0163) (Figure 8A). Moreover, rectal mDCs after the 1st Ad-SIV immunization exhibited highly significant positive correlations with rectal IFN-γ^+^ CD8^+^ memory T cells specific for SIV Gag (*r* = 0.7356, *p* = 0.0002), SIV_M766_ Env (*r* = 0.5979, *p* = 0.0054), and SIV_CG7V_ Env (*r* = 0.7615, *p* < 0.0001). Rectal mDCs also correlated significantly with SIV_M766_-specific IFN-γ^+^CD4^+^ memory T cells (*r* = 0.6033, *p* = 0.0049) and SIV_CG7V_-specific memory B cells (CD3^−^CD19^+^CD20^+^IgD^−^CD21^+/−^SIV_CG7V^+^_, *r* = 0.5977, *p* = 0.0054) (Figure 8B). Among the mDC subsets, rectal LCs after the 1st Ad-SIV immunization showed significant correlations with rectal IFN-γ^+^ CD8^+^ memory T cells specific for SIV Gag (*r* = 0.7007, *p* = 0.0006), SIV_M766_ Env (*r* = 0.6348, *p* = 0.0026), and SIV_CG7V_ Env (*r* = 0.6243, *p* = 0.0033), IFN-γ^+^CD4^+^ memory T cells specific for SIV_M766_ Env (*r* = 0.4788, *p* = 0.0327), and SIV_CG7V_-specific memory B cells (*r* = 0.4563, *p* = 0.0431) as well (Figure 8C). After the 2nd Ad-SIV immunization, the frequencies of rectal pDCs were positively correlated with SIV_CG7V_-specific IL-2^+^CD4^+^ memory T cells (*r* = 0.4658, *p* = 0.0385) (Figure 8D), the frequencies of rectal mDCs were positively correlated with SIV_CG7V_-specific memory B cells (*r* = 0.6078, *p* = 0.0075) (Figure 8E), and rectal LCs showed a positive correlation with SIV_CG7V_-specific TNF-α^+^CD4^+^ memory T cells (*r* = 0.4447, *p* = 0.0495) and SIV_CG7V_-specific memory B cells (*r* = 0.6161, *p* = 0.0065) (Figure 8F). As expected, no significant correlations were seen between SIV-specific rectal T and B cells and rectal DCs of macaques that received Ad-Empty immunizations (Supplementary Figure 3). These results support the contribution of mucosal DCs post-vaccination to development of antigen-specific adaptive immune responses in the rectal mucosa.

### Rectal DCs After Mucosal Immunization Induce Autologous Naïve T Cell Proliferation and Antigen-Specific Cytokine Production

**Figure 9:**
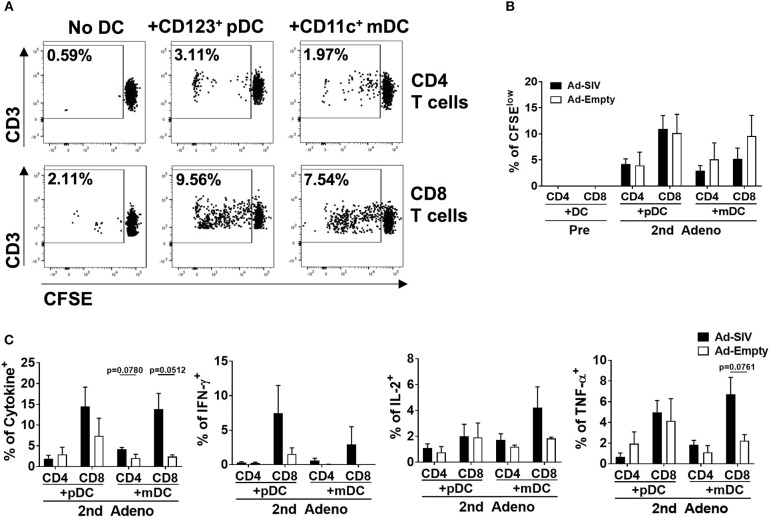
The activated rectal DCs induce autologous naïve T cell proliferation and cytokine expression. CD123^+^ pDCs and CD11c^+^ mDCs obtained from rectal tissue after the 2nd immunization were enriched and co-cultured for 6 days with autologous naïve T cells from PBMC at a DC: T cell ratio of 1:10. **(A)** The dot plots depict the gating used and the representative proportion of CFSE^low^ T cells co-cultured with Ad-SIV DCs. **(B)** Proliferation of CD4^+^ and CD8^+^ T cells after DC co-culture. Filled and empty boxes show the mean proportion of CFSE^low^ T cells co-cultured with DCs from Ad-SIV and Ad-Empty, respectively. **(C)** Frequencies of cytokine-expressing co-cultured T cells measured after 6 h of SIV_M766+CG7V_ Env peptide pool stimulation and golgi-stop treatment. Cytokine positive percentages were calculated by subtraction of unstimulated values. The left-most panel shows the % of all cytokine positive cells. Bars represent the standard error of the mean.

To evaluate rectal DC functions following immunization, we co-cultured rectal DCs with autologous naïve T cells. The naïve T cells were enriched from PBMCs which were collected before immunizations (Supplementary Figure 1A). DCs were enriched from rectal cells obtained 3 days after the 2nd immunization by using CD123 (pDCs) or CD11c (mDCs) positive magnetic sorting after eliminating T and B cells (Supplementary Figure 1B). In further CD14 and CD1c gating, the enriched pDCs showed 95.7% CD14^−^CD1c^−^ cell purity and the enriched mDCs were 91.5% CD14^−^CD1c^−^ and 6.39% CD14^−^CD1c^+^ (Supplementary Figure 1B). CD14 is expressed on the macrophage/monocyte antigen-presenting cell population. However, the fact that more than 97% of the enriched cells were CD14^−^ suggested that the majority of antigen presentation and T cell activation in the co-cultures was attributable to DCs, and that any macrophage/monocyte effects were minimal. T cell proliferation and antigen-specific cytokine production after 6-days of co-culture were assessed. Without DCs, neither CD4^+^ nor CD8^+^ T cells proliferated (Figure 9A). However, addition of rectal CD123^+^ pDCs or CD11c^+^ mDCs stimulated autologous naïve T cell proliferation (Figure 9A). CD8^+^ T cells showed greater proliferation than CD4^+^ T cells, but there was no significant difference in proliferation between Ad-SIV and Ad-Empty groups or between pDCs and mDCs (Figures 9A,B). Following stimulation of co-cultured T cells with pooled Env peptides, we measured antigen-specific cytokine production (Figure 9C). Co-culture with Ad-SIV vaccinated DCs led to greater frequencies of Env-specific IFN-γ^+^, IL-2^+^, and TNF-α^+^ CD4^+^ and CD8^+^ T cells, compared to co-culture with Ad-Empty DCs. CD8^+^ T cells from the Ad-SIV group tended to exhibit greater frequencies of cytokine^+^ cells, than CD4^+^ T cells, both individually and combined, but these differences were not significant. These data support the conclusion that the mucosal replicating Ad-SIV immunization elicited functional activation of rectal DCs with the potential to induce local and systemic antigen-specific immune responses.

## Discussion

As most HIV infections occur via mucosal transmission, strong mucosal immune responses are desirable for an efficacious HIV vaccine (36, 37). In addition to assessing mucosal immunity elicited by candidate vaccines, elucidating the mechanisms by which these immune responses develop should enhance vaccine design. As viral vectors, Ad vaccines express pathogen-associated molecular patterns easily detected by pathogen recognition receptors on innate immune cells, facilitating immune activation (10, 38, 39). Here, DCs in the rectal mucosa and blood were activated after Ad immunizations (Figures 2–5). Additionally, pDCs and LCs were significantly recruited to the rectal mucosa (Figure 2) and mDCs were increased in blood (Figure 4). It might be possible that the changes of DC frequencies were affected by alteration in cell numbers of other cell populations after immunizations, but there were no significant changes in total numbers of rectal or blood cells on which DC frequencies were based during the course of immunization. Therefore, the increases of DC frequencies after immunization were caused by DC expansion and recruitment rather than the changes in other cell populations. The lack of difference in DC frequencies or activation between Ad-SIV and Ad-Empty groups indicated that the DC recruitment and activation were induced by the Ad vector itself, rather than by antigens expressed by the vaccine. Thus, the Ad vector acted as adjuvant, inducing DC activation. Others have previously reported an adjuvant effect of Ad, attributed to the fiber protein and the hexon (40, 41).

Newly generated immature DCs migrate from bone marrow to tissues via blood. Similarly, activated DCs in local tissues migrate to LNs via blood and lymphatics. Immature mucosal DCs are immobile but are activated once they capture foreign antigens, upregulate CCR7, and gain motility. CCR7 interacts with CCL21 on lymphatic endothelial cells, providing a path for DC migration into lymphatic vessels and draining LNs. Most DC migration is via lymphatics, but pDCs can gain access to blood through high endothelial venules by means of surface adhesion molecules such as L-selectin and P-selectin glycoprotein ligand 1 (31, 42). Here, after the Ad immunizations, the frequencies of CCR7^+^ rectal pDCs and mDCs increased indicating enhanced migration potential toward LNs (Figure 3). In contrast, only CCR7^+^ pDC frequencies in blood significantly increased (Figure 5A), supporting the previous findings that migration of pDCs occurs via both lymphatics and blood, but mDCs mainly via lymphatics. However, blood mDCs were activated as shown by increased frequencies of CD86^+^, BAFF^+^, IL-6^+^, and TNF-α^+^ cells, but not CCR7^+^ cells after the 2nd Ad immunization (Figure 5B). This blood mDC activation might have been caused by systemic immune activation by the replicating Ad rather than indicating mDC migration from the mucosa.

In contrast to the significant DC recruitment and activation in rectal mucosa and blood, LN DCs did not show any changes in frequencies or activation marker expression post-immunizations except for the CCR7^+^ populations (Figures 6,7). Both rectal and blood DCs must have been migrating to LNs after their activation as shown by the increased frequencies of LN CCR7^+^ DCs. However, it's not clear why LN DCs did not show increased activation characteristics. The time of peak DC activation may have been earlier than 14 days post-immunization. In mice, once tissue-derived DCs arrive in draining LNs after activation, the survival time of mature DCs within the lymphoid organs is only 2–9 days (43, 44). Moreover, the frequencies of CD40^+^ and CD86^+^ LN DCs before immunization were already higher than those in rectal and blood DCs. Due to the highly activated environment, detection of LN changes post-immunizations may have been difficult.

Mucosal DCs deliver and present antigenic information to naïve T cells in LNs, but they are also involved in T cell homing from the lymphoid organ to the mucosa after T cell activation. DCs from intestinal tissues induce expression of gut homing markers such as α4β7 and CCR9 on T and B cells (34, 35, 45, 46). Here we found significant correlations between DCs and antigen-specific memory T and B cells after Ad-SIV immunization in rectal tissue (Figure 8), suggesting that mucosal DC activation was critical for induction of mucosal memory T and B cell responses after Ad immunization. The fact that most significant correlations were found after the 1st Ad immunization might be due to the different mucosal immunization routes. Intranasal immunization induces antigen-specific immune responses in the upper and lower respiratory, gastric, and genital tracts, while oral immunization affects the gastrointestinal tract and mammary glands (37, 47). The 1st intranasal and oral replicating Ad immunization delivered antigen to the rectal mucosa, contributing directly to DC activation and memory T and B cell responses. However, the 2nd replicating Ad immunization was given intratracheally, so immune responses in the rectal mucosa were more likely due to indirect systemic immune activation rather than direct antigen exposure, even though much stronger DC activation was induced in the rectal mucosa (Figure 3). The strong DC activation responses after the 2nd Ad seemed more innate and pro-inflammatory than adaptive.

Using an *in vitro* co-culture system, we demonstrated that rectal DCs were functionally activated after Ad immunizations and induced autologous naïve T cell activation (Figure 9). CD8^+^ T cells were more proliferative than CD4^+^ T cells after co-culture with DCs, and there was no difference between pDC and mDC in inducing T cell proliferation. Because frequencies of activated rectal DCs from the Ad-Empty group were increased after immunization, it was not surprising that they induced some T cell proliferation. Nevertheless, after 6-days of co-culture and 6 h of Env peptide pool stimulation, T cells co-cultured with DCs from Ad-SIV-immunized macaques showed enhanced frequencies of cytokine^+^ cells approaching significance compared to those co-cultured with DCs from macaques that received Ad-Empty vectors (Figure 9C). These results clearly indicate that the activated rectal DCs, both pDCs and mDCs, were capable of inducing antigen-specific T cell proliferation and cytokine production.

Taken together, our results show that mucosal Ad immunizations could recruit and activate DCs in rectal mucosa and blood and induce DC migration to draining lymph nodes. In addition, we showed that the activated mucosal DCs play a role in inducing antigen-specific T cell proliferation and cytokine production. Our findings identify the initial cellular mechanisms of the replicating Ad vaccine regimen in the rhesus macaque model and highlight the rapid response and potential roles of DCs in mucosal immune activation after replicating Ad immunizations. This information is valuable for development of HIV vaccines with enhanced efficacy against mucosal transmission and for improved mucosal vaccines in general.

## Ethics Statement

This study was carried out in accordance with the guidelines of the Association for the Assessment and Accreditation of Laboratory Animal Care. The protocol and procedures were approved by the National Cancer Institute Animal Care and Use Committee.

## Author Contributions

E-JK and MR-G developed the concept and designed the experimental plans. E-JK, SH, GE-A, MR, and TH performed experiments. E-JK analyzed data. E-JK and MR-G prepared the manuscript. All authors reviewed the manuscript.

### Conflict of Interest Statement

The authors declare that the research was conducted in the absence of any commercial or financial relationships that could be construed as a potential conflict of interest.
