# Supplementary material for: Replicating Adenovirus-SIV Immunization of Rhesus Macaques Induces Mucosal Dendritic Cell Activation and Function Leading to Rectal Immune Responses
Source: Front Immunol. 2019 Apr 12;10:779. doi: 10.3389/fimmu.2019.00779 (PMC6473464; doi:10.3389/fimmu.2019.00779)
Supplement: Supplementary file 1 [file Data_Sheet_1.PDF]

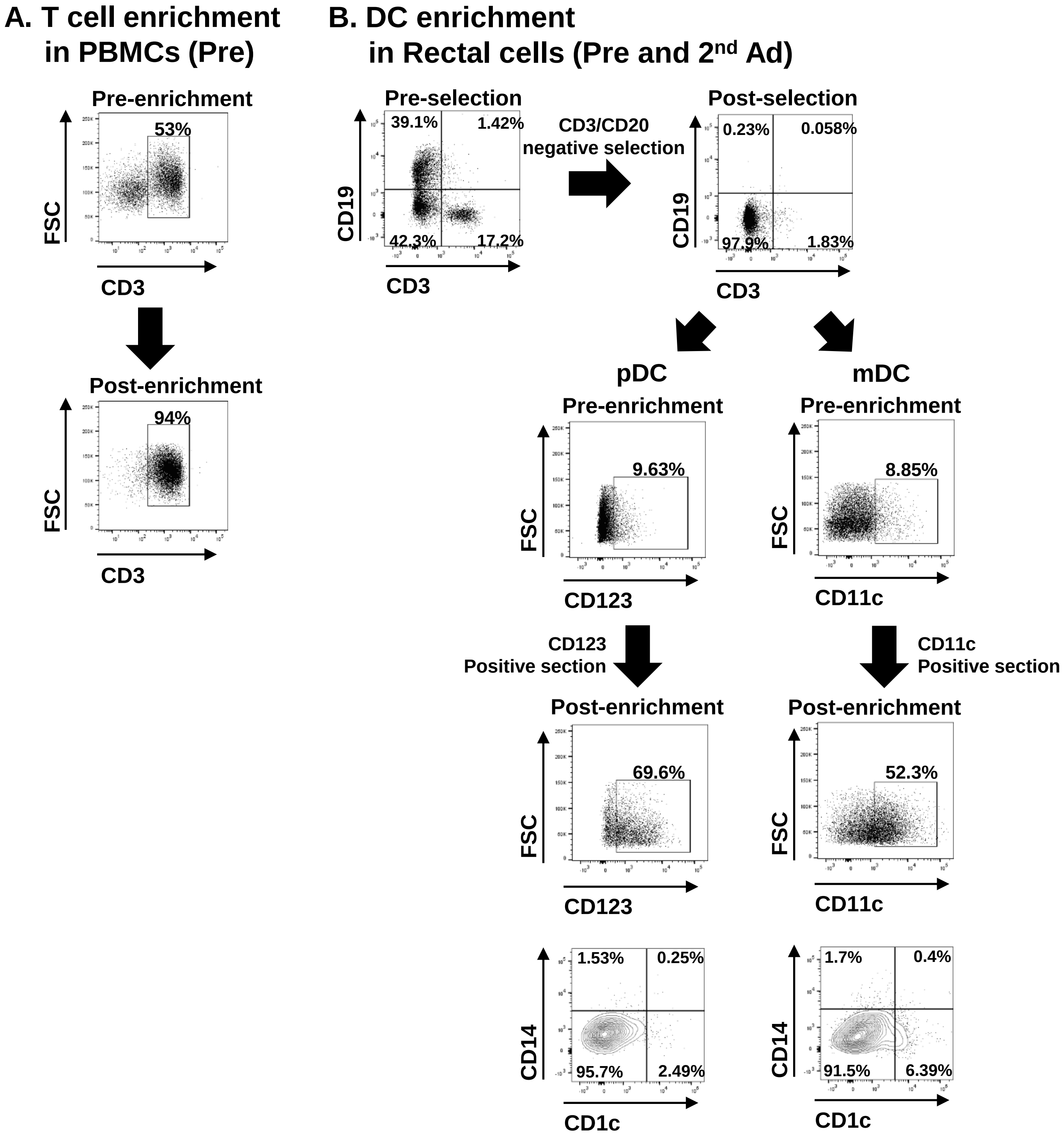

Supplementary figure 1. T cell and DC enrichment for co-culture. Magnetic sorting was used to enrich the cells. **(A)** Naïve T cells from pre-immunization PBMCs were enriched. Representative CD3 positive populations pre- and post-enrichment are shown. **(B)** Rectal DCs were enriched from pre- and post 2nd Ad samples. First, CD3 and CD19 positive cells were excluded. Then CD123 or CD11c positive selection was performed. Further analysis showed minimal CD14^{+} cell contamination in the enriched populations. A representative example is shown.

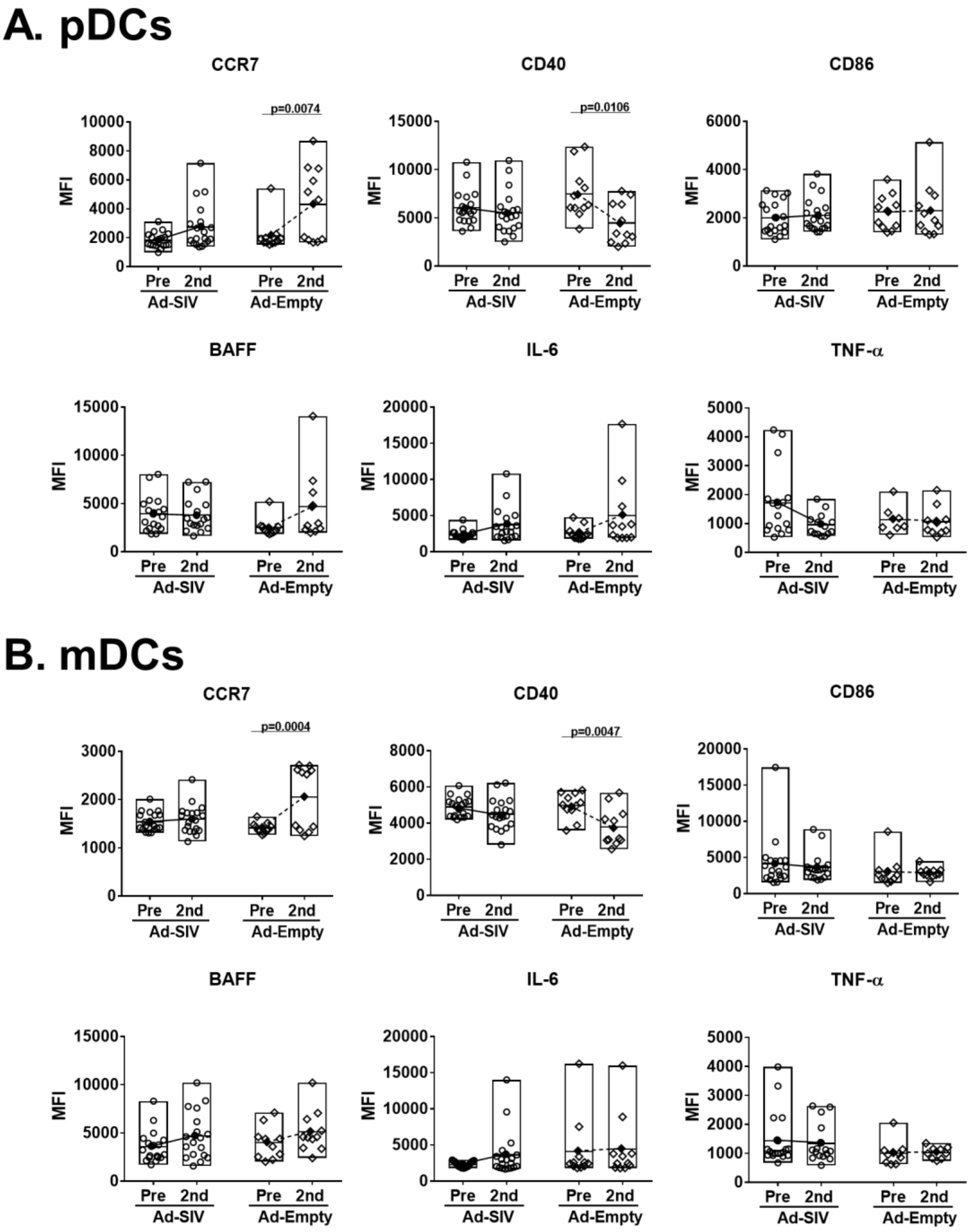

Supplementary figure 2. Activation marker and cytokine expression of LN DCs. Mean fluorescence intensity (MFI) of each activation marker and cytokine on pDC (**A**) and mDC (**B**) cells is shown. The open and filled dots show the individual and mean values, respectively. For statistical analysis, two-way ANOVA and Tukey post-multiple comparison test were performed.

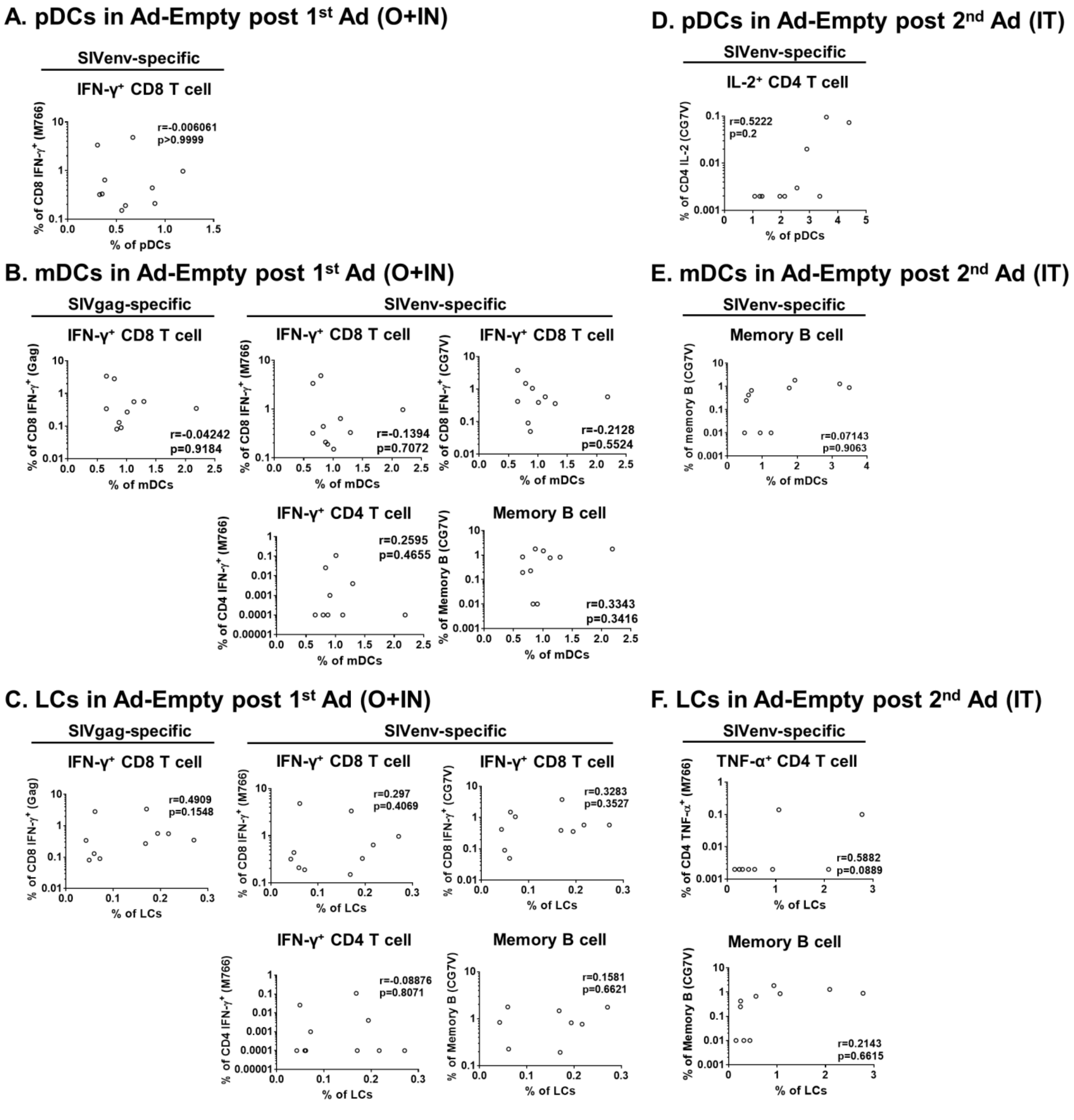

Supplementary Figure 3. DC frequencies in Ad-Empty immunized group have no correlation with antigen-specific T and B cell responses in rectal mucosa. Nonparametric Spearman test was performed for correlation analysis by using the data obtained after 1st Ad-Empty (**A-C**) and 2nd Ad-Empty (**D-F**) immunizations.
